# DNTB: Dual-branch network model based on transformer and Bi-LSTM for energy consumption prediction in building chiller systems

**DOI:** 10.1371/journal.pone.0330187

**Published:** 2025-10-03

**Authors:** Andong Chen, Mingtao Wu, Cheng Chen, Chen Chen, Yong Huang, Xiaoyi Lv

**Affiliations:** 1 Jiangsu Railway Group Rongfa Management Co., Ltd., Nanjing, Jiangsu, China; 2 School of Computer Science and Technology, Xinjiang University, Urumqi, Xinjiang, China; 3 School of Software, Xinjiang University, Urumqi, Xinjiang, China; 4 Xinjiang Clinical Research Center for Rheumatoid Arthritis, Urumqi, Xinjiang, China; 5 Xinjiang Medical University, Urumqi, Xinjiang, China; 6 School of Civil Engineering, Xinjiang University, Urumqi, Xinjiang, China; 7 Key Laboratory of Signal Detection and Processing, Xinjiang University, Urumqi, Xinjiang, China; Aalto University, FINLAND

## Abstract

Accurate prediction of chiller energy consumption is crucial for reducing building energy consumption. In this study, an innovative dual-branch network architecture DNTB (A Dual-Branch Network Model Based on Transformer and Bi-LSTM for Energy Consumption Prediction in Building Chiller Systems) was proposed to address the problems of insufficient long-term dependency modeling and noise sensitivity in current prediction models. The research goal is to develop a prediction model that can simultaneously process temporal features and global dependencies. The basic principle is to utilize the complementary characteristics of Transformer and Bi-LSTM. Transformer is sensitive to data noise and Bi-LSTM is weak in capturing long-term sequence information. It can better capture the temporal information of chiller energy consumption data and well model the relationship between variables such as chilled water, building load, chiller temperature, humidity, dew point and chiller energy consumption. In order to prove the effectiveness and generalization ability of the model, experiments were carried out on long-term and short-term tasks of chiller energy consumption prediction. The long-term prediction results had MSE (mean absolute error) of 0.0051, RMSE (mean square error) of 0.0605, and R2 (coefficient of determination) of 0.8031. The short-term prediction results had MSE of 0.0080, RMSE of 0.0738, and R2 of 0.6717. The experimental results indicate that DNTB performs excellently in both long-term and short-term chiller energy consumption prediction, making it a robust framework for chiller energy consumption prediction. The introduction of DNTB enriches the diversity of empirical model algorithms.

## 1. Introduction

In recent years, global warming and the greenhouse effect have become significant challenges worldwide [1]. To address the urgent need to combat climate change, governments and international organizations have proposed carbon neutrality goals [2,3].

In the building sector, reducing energy consumption and carbon emissions is critical to achieving carbon neutrality. With the growing demand for air conditioning and cooling systems, the energy consumption of Heating, Ventilation, and Air Conditioning (HVAC) systems, particularly chillers, has become a significant component of total building energy consumption. Chillers consume substantial amounts of energy; therefore, optimizing chiller energy usage is a vital measure to reduce building electricity consumption. Accurate chiller energy consumption predictions can assist building managers in making efficient and informed decisions to reduce energy waste [4]. However, chillers exhibit characteristics such as lag effects, nonlinearity, and strong coupling, which make energy optimization costly. These factors pose significant challenges to effectively predicting and optimizing chiller energy consumption in practical applications. Consequently, establishing an accurate prediction model for chiller energy consumption under various conditions is key to achieving energy savings in buildings.

In the early stages of energy prediction research, traditional statistical and physical models were widely used [5,6]. These studies primarily relied on empirical formulas and energy consumption models. After 2000, with advancements in computational power, machine learning was introduced into various energy prediction fields. For instance, Fang et al. (2016) employed a multivariate regression model to forecast energy consumption in district heating systems [7]. Support Vector Regression (SVR) effectively addressed the nonlinearity in chiller energy prediction by constructing hyperplanes in high-dimensional space to model the relationship between energy consumption and environmental factors. Similarly, Becker et al. (2017) applied K-Nearest Neighbors (KNN) and Random Forest algorithms to predict wind energy consumption in power systems [8]. KNN, a simple and intuitive non-parametric method, predicted future chiller energy consumption based on the similarity of historical data, while Random Forest used tree-structured models to capture complex energy consumption patterns and model nonlinear relationships between features.

Post-2010, with the advent of big data and further improvements in computational power, deep neural network methods became widely applied to energy consumption prediction. Artificial Neural Networks (ANNs), inspired by human biological neural networks, are nonlinear statistical methods capable of predicting various forms of building energy consumption, including total energy use, heating and cooling loads, and electricity consumption. Deep learning has made significant progress in the field of time series prediction. Many studies [9–15] have shown that the hybrid neural network architecture has demonstrated excellent performance in complex system modeling such as stock price prediction (Zhou & Wu, 2024), oil and gas engineering parameter prediction (Alakbari et al., 2021, 2023), and rock mechanics analysis (Alakbari et al., 2024), which provides important theoretical support for the construction of a dual-branch prediction model in this study. Two primary categories of ANN methods—backpropagation neural networks and generalized regression neural networks—have also been applied to energy consumption prediction [16,17]. However, despite these algorithms laying a foundation for chiller energy consumption prediction, traditional machine learning or deep learning models have certain limitations, particularly when dealing with complex dynamic systems and large datasets. These algorithms often suffer from strong dependencies on feature engineering, insufficient temporal modeling capabilities, and poor generalization performance.

In recent years, artificial intelligence combined with big data analytics has increasingly gained attention in building energy prediction [18–22]. Research in this field spans areas such as chiller energy consumption prediction [23–27], fault detection in building chillers [26], and predictive control optimization [4,28–31]. Traditional machine learning and deep learning approaches no longer suffice for building energy consumption predictions. Long Short-Term Memory (LSTM) networks, designed to address the long-distance dependency problem in traditional Recurrent Neural Networks (RNNs), utilize memory cells and three gating mechanisms (input, forget, and output gates) to selectively retain or discard information [32]. Bi-Directional LSTM (Bi-LSTM), an extension of LSTM, processes sequential data from both forward and backward directions, capturing contextual information. It is widely applied in sequence prediction and time series analysis tasks [33]. Bi-LSTM can effectively model the complex nonlinear relationships in energy consumption prediction, making it suitable for real-time and non-real-time forecasting. Numerous studies have demonstrated the advantages of LSTM in HVAC energy consumption prediction. For instance, F. Mtibaa et al. proposed LSTM-MISO and LSTM-MIMO models, which were evaluated using real-world case studies of buildings employing variable air volume (VAV) and constant air volume (CAV) systems. Results showed that the LSTM model outperformed multilayer perceptron models, reducing prediction errors by 50% [34]. Similarly, MJ Ellis et al. developed an encoder-decoder LSTM-based EMPC framework, which pre-cooled building thermal zones and reduced operational costs compared to maximum temperature maintenance approaches [35]. Transformer models, based on attention mechanisms, represent another milestone in deep learning. Initially introduced by Vaswani et al. in 2017 for natural language processing (NLP) tasks [36], Transformers leverage self-attention mechanisms and encoder-decoder architectures to process sequential data efficiently while addressing the long-distance dependency problems inherent in traditional RNNs. With strong temporal modeling capabilities, parallel computation efficiency, and the ability to capture complex dependencies, Transformers have become powerful tools in energy prediction, especially for tasks requiring multi-feature and long-sequence dependencies in chiller energy consumption prediction. For example, LIM B et al. achieved remarkable performance using Transformers for forecasting in power load, traffic, retail, and stock domains [37]. Similarly, Long Li et al. proposed a Transformer-based model for building cooling load prediction, improving load forecasting accuracy [38]. Despite their potential, Transformer and Bi-LSTM models have limitations when applied to chiller energy consumption prediction. Bi-LSTM struggles to capture long-term dependencies, such as seasonal factors affecting energy consumption. Additionally, Bi-LSTM may face training difficulties due to gradient vanishing or exploding issues. On the other hand, Transformers are sensitive to data quality and noise, which can negatively impact performance when dealing with noisy or anomalous data. The theoretical basis of this study lies in two points. First, the self-attention mechanism of Transformer can effectively model the global dependency between variables, but it requires high-quality data support. Second, the bidirectional time series processing capability of Bi-LSTM is good at capturing local time patterns, but its ability to model long-term trends is limited.

Given these research advancements and the limitations of existing models, this study develops a dual-branch network based on Transformer and Bi-LSTM (DNTB) for efficient prediction of chiller energy consumption in building energy systems. The main innovations of this study include:

1. Proposing a dual-branch structure that leverages the complementary advantages of Transformer and Bi-LSTM in capturing long-term and short-term dependencies in sequential data, enabling accurate chiller energy consumption prediction.
2. Introducing a decision-level fusion module that integrates the complementary information from the two branches through averaging their output representations to derive the final prediction.
3. This study proposes a deep learning-based model named DNTB (Dual-branch Network based on Transformer and Bi-LSTM) that achieves superior performance in long-term and short-term chiller energy consumption prediction tasks.

## 2. Method

**Fig 1:**
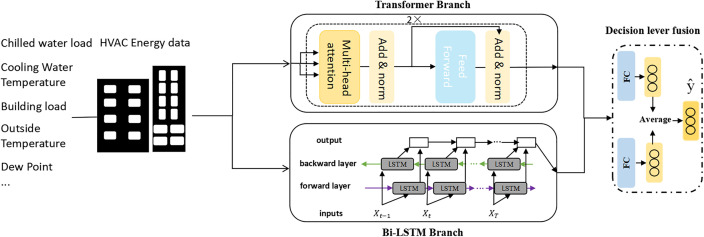
Framework diagram of the proposed model.

The Dual-branch Network based on Transformer and Bi-LSTM (DNTB) is an innovative and efficient framework for predicting chiller energy consumption. It boasts high accuracy and robust generalization capability. The framework is illustrated in Fig 1. First, sensor data collected from the chiller system is input into the framework. The raw chiller data is processed through two branches: a Transformer branch and a Bi-LSTM branch. The Transformer branch extracts important features and sequential information from the raw data and outputs corresponding representations. Simultaneously, the Bi-LSTM branch captures the dynamic temporal features of the chiller’s time series data from both forward and backward directions, producing sequence-aware representations. Finally, these representations, which contain extracted sequential information and high-level features, are input into a decision-level fusion module. After being passed through individual fully connected layers, the outputs from the two branches are averaged to yield the final prediction. The detailed structure of DNTB is explained below.

### 2.1. Transformer branch

In the standard Transformer architecture, there are two main components: the encoder and the decoder [36]. The encoder processes input sequences and transforms them into hidden state vectors, extracting features and contextual information to generate high-dimensional representations of the input data. The decoder typically generates output sequences (e.g., translating from one language to another). However, in chiller energy consumption prediction tasks, the goal is not to generate an output sequence but to predict a single value. Thus, the proposed model employs only the encoder component of the Transformer to produce sequence-aware representations.

Initially, sequential input data is mapped to vectors of a specified dimension and fed into the Transformer’s encoder. The encoder comprises four components: a multi-head attention mechanism, a normalization layer, a feedforward neural network layer, and another normalization layer. These components are detailed below:

#### Multi-head attention mechanism.

This component is composed of multiple attention layers. The attention mechanism (AM), a core concept in the Transformer, mimics how the human brain focuses on specific information. It scans the target region quickly, identifying relevant features. The attention mechanism is based on scaled dot-product attention, which maps a query (Q) and a set of key (K) and value (V) pairs to an output. The data received are either the input or output of the previous encoder, and weight matrices are applied to compute Q, K, and V. The computation is as follows:


$$Query=W_{Q}X_{i}$$ (1)

$$Key=W_{K}X_{i}$$ (2)

$$Value=W_{V}X_{i}$$ (3)

$$Attention(Query,Key,Value)=Softmax(\frac{\left(Query,\;{Key}^{T}\right)}{\sqrt{d_{k}}})$$ (4)

where $W_{Q}$, $W_{K}$, and $W_{V}$ represent the weight matrices used to calculate Query, Key, and Value, respectively. $X_{i}$ is the input variable. Q, K, and V represent the query, key, and value matrices, respectively. $d_{k}$ is a scaling factor to adjust the attention scores. The multi-head attention mechanism stacks several attention layers, with each layer capturing features from different subspaces. This enables the model to extract more comprehensive features compared to single-head attention.

#### Add & normalize layer.

After extracting temporal features via multi-head attention, the output is normalized to enhance the model’s robustness. The normalization formula is given by:


$${f(x)}_{Add\&Normalize}=LayerNorm\left(x+Sublayer(x)\right)$$ (5)

#### Feedforward Neural Network (FFN).

The FFN consists of two linear transformations with a ReLU activation function, which enhances the model’s non-linear fitting capability. The FFN transforms the input vector before passing it to the next module:


$$FNN(x)=W_{\text{2}}(\text{max}\left(\text{0},\;W_{\text{1}}x+\;b_{\text{1}}\right))+b_{\text{2}}$$ (6)

Here, $W_{\text{1}}$, $W_{\text{2}}$, $\;b_{\text{1}}$, and $b_{\text{2}}$ represent the weights and biases of the FFN layers.

#### Residual connection and normalization.

The final output of the FFN undergoes residual connection and normalization to further improve robustness.

After processing through these layers, the Transformer branch outputs a sequence-aware representation.

### 2.2. Bi-LSTM branch

Recurrent Neural Networks (RNNs) are designed specifically for sequence and time-dependent predictions. LSTM (Long Short-Term Memory) networks address the issue of long-term dependency in traditional RNNs [39]. LSTM has shown superior performance in chiller energy consumption prediction compared to traditional models like linear regression and support vector machines. LSTM can capture temporal dependencies, handle nonlinearity, and reduce the interference of noise, making it particularly suitable for short-term energy prediction tasks.

The core components of LSTM include a memory cell and three gating mechanisms: the forget gate, input gate, and output gate. These mechanisms control the selective retention and forgetting of information. The following describes their operations:

**Forget Gate**: Determines which information to discard from the cell state:


$$f_{t}=\sigma (W_{f}\cdot \left[h_{t-\text{1}},x_{t}\right]+b_{f})$$ (7)

**Input Gate**: Decides which information to update in the cell state:


$$i_{t}=\sigma (W_{i}\cdot \left[h_{t-\text{1}},x_{t}\right]+b_{i})$$ (8)

$$\tilde{C_{t}}=tanh(W_{C}\cdot \left[h_{t-\text{1}},x_{t}\right]+b_{C})$$ (9)

**Output Gate:** Determines the output based on the updated cell state:


$$o_{t}=\sigma \left(W_{o}\cdot \left[h_{t-\text{1}},x_{t}\right]+b_{o}\right)$$ (10)

**State Updates:** The cell state and hidden state are updated as follows:


$$C_{t}=f_{t}\cdot C_{t-\text{1}}+i_{t}\cdot \tilde{C_{t}}$$ (11)

$$h_{t}=o_{t}\cdot \text{tanh}(C_{t})$$ (12)

Among them, σ() is the sigmoid function, $W_{f}$, $W_{i}$,$W_{O}$ are weight matrices used to update the states of the forget gate, input gate, and output gate, $b_{f}$, $b_{i}$, $b_{o}$ are bias vectors used to update the states of the forget gate, input gate, and output gate, respectively. $h_{t}$ is the activation value at time step $h_{t-\text{1}}$, $x_{t}$ is the input of the time step, $C_{t}$ is the memory candidate of the unit time step t, tanh() is the activation function, $W_{C}$ is the weight matrix used to calculate the memory candidate, and $b_{C}$ is the bias vector used to update the memory candidate. h_t is the activation value at time step $h_{t-\text{1}}$. For Bi-LSTM, the forward and reverse hidden states are concatenated at each time step to form the final output. In our model, the original chiller data is input as the input of the Bi-LSTM branch, the time series of the chiller data is analyzed, and finally a representation $V_{B}$ with a good time series is output.

### 2.3. Decision level fusion

After the previous Transformer branch and Bi-LSTM branch processing, two feature representations $V_{T}$ and $V_{B}$ with sequence information are obtained respectively. Although they both contain sequence feature information, they are obtained using different sequence feature extraction methods, so there is heterogeneity between them. Therefore, how to fuse the output representations of the two branches well is particularly critical. In this study, we use decision-level fusion. Common decision fusion methods include averaging [40], majority voting [41], weighted and learnable models [42], etc. This study uses average decision-level fusion, which can effectively retain the effective information in each branch and reduce the risk of overfitting. In this section, the two representations $V_{T}$ and $V_{B}$ will be passed through their respective fully connected layers to obtain two regression result decisions, and then the two classification decisions will be averaged at the decision level to finally obtain the prediction result. The following will introduce the internal structure of decision-level fusion in detail.

First, the two input representations $V_{T}$ and $V_{B}$ are subjected to dropout regularization, which does not change the shape of the tensor. In order to achieve nonlinear transformation, a fully connected layer with Gelu as the activation function is used. The formula of the GeLU activation function refers to (13). Finally, two regression result decisions $P_{T}$ and $P_{B}$ are output. Among them, $P_{T}$ is the regression result decision of the Transformer branch, and $P_{B}$ is the regression result decision of the Bi-LSTM. Finally, the two regression result decisions are averaged and fused to obtain $\hat{y}$. The mathematical formula of the relevant operation is as follows:


$$GeLU(x)=\text{0}.\text{5}x(\text{1}+\text{tanh}(\sqrt{\frac{\text{2}}{\Pi }}(x+\text{0}.\text{044715}x^{\text{3}})))$$ (13)

$$P_{T}=GeLU\left(W_{T}V_{T}\;+\;b_{T}\right)$$ (14)

$$P_{B}=GeLU(W_{B}V_{B}\;+\;b_{B})$$ (15)

$$\hat{y}=\;\frac{P_{T}+P_{B}}{\text{2}}$$ (16)

Among them, $W_{T}$, $W_{B}$ are the weight matrices of the linear layer, $b_{T}$, $b_{B}$ are the bias vectors of the linear layer. $\hat{y}\;$is the final prediction result.

### 2.4. Training setup

Hyperparameter tuning of the model is crucial to achieve the best prediction performance. The specific Python version libraries used are shown in Table 1. Through a large number of experimental tests and comparisons, we obtained the parameters and hyperparameters based on the DNTB model, as shown in the table. In all experiments, the Adam optimizer was used uniformly, and the learning rate was uniformly set to 0.001. There are slight differences between long-term and short-term predictions in some parameters. For example, in the long-term prediction of chiller energy consumption, epochs are set to 100, Sequence length is set to 4, and hidden size is set to 64. In the short-term prediction of chillers, epochs are set to 150, Sequence length is set to 1, and hidden size is set to 32. Appropriate parameters can allow the model to fit the energy consumption curve trend better and faster. For specific parameters, see Table 2. The long-term task is set to 8 heads and the short-term task is set to 4 heads. The main purpose is that the long-term task requires more heads to model global dependencies, while the short-term task can reduce redundant calculations with fewer heads. The Bi-LSTM hidden size is set to 64 for the long-term task and 32 for the short-term task. The purpose is that the long-term task requires a larger capacity to store bidirectional time series information, while the short-term task avoids overfitting by compressing the dimension. During the training process, we use mean square error as the loss function to train the model:

**Table 1:** The versions and functions of the key Python function libraries used.

Library	Version	Function
Torch	2.1.1+cu118	PyTorch Framework
Transformers	4.2.1	Provides pre-trained Transformer architecture and tools
Numpy	1.26.3	Numerical computing basic library
Pandas	1.2.4	Data processing and analysis
Scikit-learn	0.24.2	Data preprocessing and model evaluation
Matplotlib	3.5.1	Visualization
Seaborn	0.11.2	Data distribution visualization

**Table 2:** The setting of Hyper parameter.

Hyper-parameter	Value Long-term task	Value Short-term task
Batch size	128	256
Optimizer	Adam	Adam
Epochs	100	150
Dropout	0.04	0.08
Learning rate	0.001	0.001
Sequence length	8	1
Embedding size for each token (Transformer branch)	64	32
Number of attention head (Transformer branch)	8	4
Dim of feed forward (Transformer branch)	64	32
Number of Layer (Bi-LSTM)	2	2
Input size (Bi-LSTM)	64	32
Hidden size (Bi-LSTM)	64	32


$$MSE=\;\frac{\text{1}}{n}\sum_{i=\text{1}}^{n}{\left(y_{i}-{\hat{y}}_{i}\right)}^{\text{2}}$$ (17)

**Fig 2:**
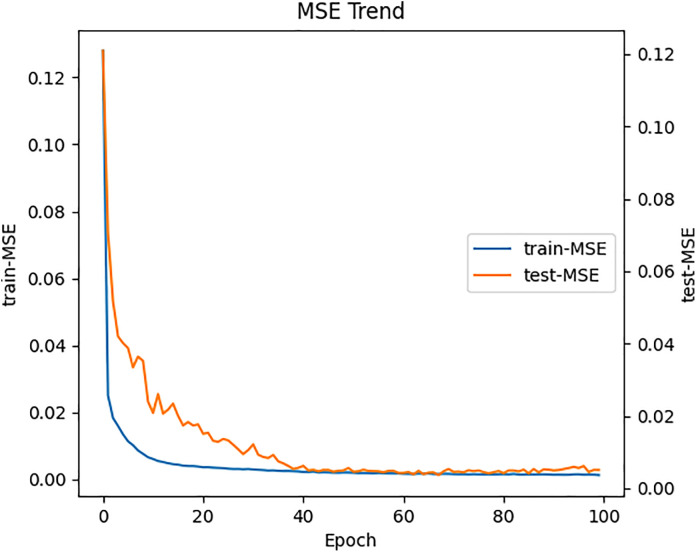
The loss convergence curves of training and testing on the long-term prediction task of chiller energy consumption.

**Fig 3:**
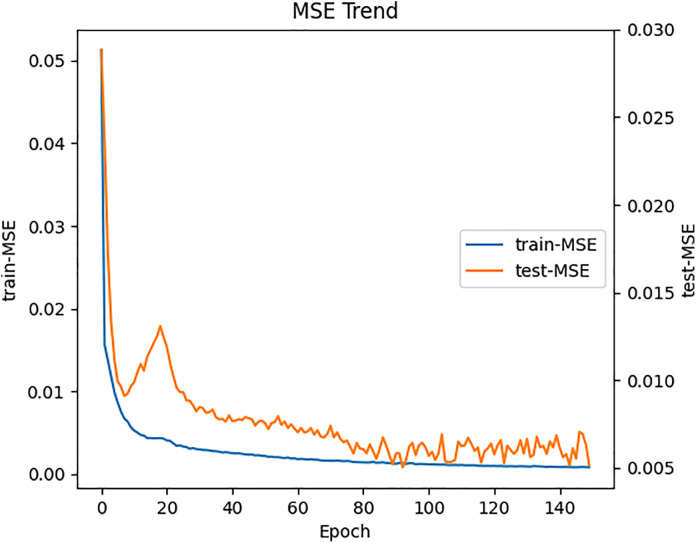
The loss convergence curves of training and testing on the short-term prediction task of chiller energy consumption.

The model was implemented using the PyTorch framework, a mature Python neural network framework, and the NVIDIA A40 GPU was used on Windows to implement a dual-branch network based on Transformer and Bi-LSTM for efficient prediction of building energy chillers. The loss values of the model training and testing for chiller energy consumption prediction converge as shown in Figs 2 and 3. We believe that the experimental parameters are set so that the DNTB model fits the chiller energy consumption data well.

### 2.5. Baseline

To validate the effectiveness and generalization capability of the proposed DNTB model, this study selected several state-of-the-art baseline models from the field of deep learning, particularly those widely used in time-series data analysis. The selected baseline models include Bi-LSTM, Transformer, AlexNet, ResNet, and VGG-19. A brief description of each baseline model is provided below:

**Bi-LSTM** [39]: Proposed by Hochreiter and Schmidhuber in 1997, Bi-LSTM (Bidirectional Long Short-Term Memory) has undergone significant advancements in subsequent research. It is a powerful sequential modeling tool, performing exceptionally well in processing time-series data and natural language processing tasks.

**AlexNet** [43]: AlexNet, introduced by Alex Krizhevsky et al. in 2012, marked a major breakthrough in deep learning for computer vision. It was the first convolutional neural network (CNN) to achieve significant performance improvements on the ImageNet dataset. AlexNet consists of multiple convolutional layers, pooling layers, and fully connected layers. It employs Dropout and ReLU activation functions, laying the foundation for modern CNN-based image classification models.

**VGG-19** [44]: VGG-19, proposed by Karen Simonyan and Andrew Zisserman in 2014, is a deep convolutional neural network known for its simplicity and effectiveness. It demonstrated remarkable performance on the ImageNet dataset by utilizing small convolutional kernels and a consistent network architecture.

**ResNet** [45]: ResNet (Residual Network), introduced by Kaiming He et al. in 2015, achieved groundbreaking performance on the ImageNet dataset. The core innovation of ResNet is its residual connections, which allow the network to learn residual functions instead of direct mappings. This design addresses the gradient vanishing and exploding problems often encountered in deep networks, enabling the training of very deep models.

**Transformer** [36]: Transformer, proposed by Vaswani et al. in 2017, leverages self-attention mechanisms to capture long-range dependencies in sequential data. The multi-head attention mechanism in Transformer allows the model to learn multiple representations of the sequence simultaneously, making it highly effective for tasks involving complex sequential relationships. Transformers have shown significant success in NLP tasks such as machine translation, text generation, and question answering.

**TCN** [46]: TCN (Temporal Convolutional Network) is a time series modeling method based on convolutional neural networks. It captures long-term dependencies through dilated causal convolution and residual connections. Unlike traditional recurrent neural networks (RNNs), TCN uses a convolutional structure to achieve parallel computing, avoiding the time overhead of recursive computing. At the same time, it expands the receptive field in the time dimension through dilated convolution, which can effectively model long-distance temporal dependencies. Its causality ensures that the prediction depends only on historical data. It is suitable for single-step and multi-step time series prediction tasks and performs well in energy consumption prediction, speech recognition and other fields.

**Informer** [47]: Informer is an improved time series prediction model based on Transformer. To address the high computational complexity and memory consumption of traditional Transformer in long sequence prediction, Prob Sparse Self-Attention and Distilling Operation are proposed. Prob Sparse Attention reduces the amount of computation by screening key time points, while the distillation operation reduces the sequence length layer by layer, further improving the efficiency of long sequence modeling. While maintaining the global dependency modeling capability of Transformer, Informer significantly improves the practicality of long time series prediction and is widely used in scenarios that require processing of ultra-long input sequences, such as power load forecasting and meteorological data modeling.

The performance of these baseline models is compared to the DNTB model in both long-term and short-term chiller energy consumption prediction tasks, as discussed in subsequent sections.

## 3. Data analysis

The dataset used in this study was obtained from Kaggle and consists of chiller energy consumption data from commercial buildings in Singapore. The dataset used in this article is provided as an attachment. The time span of the dataset ranges from August 18, 2019, 00:00, to June 1, 2020, 13:00 [48]. Data collection was performed at 30-minute intervals, resulting in a total of 13,615 records. Then, we preprocessed the data, including removing outliers and using the mean value to interpolate missing values. The outlier screening used a sliding window (IQR) method to eliminate abnormal data, and finally obtained 13,610 data as experimental samples. This robust dataset provided a solid foundation for evaluating the proposed DNTB model, ensuring accuracy and reliability in chiller energy consumption prediction.

Subsequently, a Spearman correlation analysis was conducted to examine the relationships between the dataset’s variables and chiller energy consumption (Chiller Consumption, measured in kWh). The variables analyzed included Chilled Water Rate (L/sec), Cooling Water Temperature (°C), Building Load (RT), Outside Temperature (°F), Dew Point (°F), Humidity (%), Wind Speed (mph), and Pressure (in). The Spearman correlation coefficient is a statistical measure that evaluates the monotonic relationship (not necessarily linear) between two variables. Its values range from –1 to 1, where −1 indicates a perfect negative correlation, 1 indicates a perfect positive correlation, and 0 suggests no correlation [49]. In this study, we divide the correlation into three levels. The absolute value of the Spearman correlation coefficient is less than 0.45, which means the correlation is weak. The absolute value of the Spearman correlation coefficient is greater than or equal to 0.45 and less than 0.5, which means the correlation is strong. The absolute value of the Spearman correlation coefficient is greater than or equal to 0.5, which means the correlation is very strong. The experimental results are shown in Table 3. It can be seen from the table that there are five variables that have a very strong correlation with Chiller Consumption, namely Chilled Water Rate, Colling Water Temperature, Building Load, Outside Temperature and Humidity. Among them, Building Load has the strongest correlation with Chilled Consumption, with a Spearman value of 0.903364343, which indicates that when the Chilled Water Rate increases, the energy consumption of the chiller will increase. It is worth noting that the correlation coefficient between Humidity and Chiller Consumption is −0.589924807, which indicates that when Humidity increases, the energy consumption of the chiller will decrease. These correlation experiments provide a theoretical basis for using these different variables to predict the energy consumption of chillers.

**Table 3:** Correlation between chiller consumption and other variables.

Variable	Correlation
Chilled Water Rate	0.829545171
Cooling Water Temperature	0.560194975
Building Load	0.903364343
Outside Temperature	0.634466933
Dew Point	−0.107909838
Humidity	−0.589924807
Wind Speed	0.45794891
Pressure	−0.135400447

**Fig 4:**
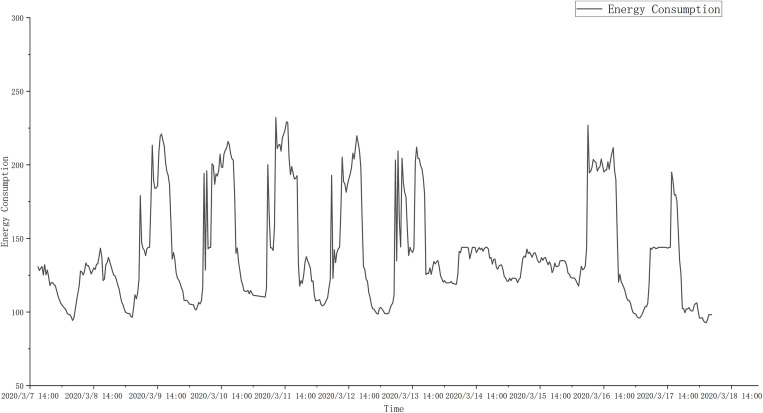
Test set chiller energy consumption curve for a period of time.

In this section, we will introduce the partitioning of the chiller dataset. The prediction tasks are long-term prediction and short-term prediction. Depending on the task, the dataset is partitioned in different ways. First, for long-term prediction, in order to address the time series nature, the dataset is partitioned in chronological order to ensure that the training subset contains the earlier time period and the test set contains the later time period. This dataset partitioning strategy enables the model to predict future results based on historical patterns. The dataset is divided into a 70% training subset and a 30% test subset, which contain 9530 training samples and 4080 test samples respectively. In short-term prediction, the dataset is partitioned in chronological order using a five-fold cross-validation split method. The dataset is divided into an 80% training subset and a 20% test subset, which contain 10890 training instances and 2720 test instances respectively. The five-fold cross-validation method helps to reduce overfitting and bias and improve the generalization ability of the model [50]. Fig 4 shows the curve of the chiller energy consumption forecast for the test set from March 7 to March 18, 2020 in the long-term forecast. Repeated troughs and unique peaks appear in the image, indicating the fluctuation of the chiller energy consumption curve. There are many reasons for the troughs in the energy consumption data, including low building load (RT), changes in external temperature, or periods of reduced building occupancy, resulting in reduced chiller energy use. Similarly, high peaks mean the opposite, such as high building loads during the morning working peak, increased building occupancy, etc. These fluctuations are also affected by other factors such as humidity levels and chilled water flow, resulting in overall changes in the data.

## 4. Experimental and discussion of result

### 4.1. Model evaluation indexes

To quantitatively evaluate the proposed model for chiller energy consumption prediction, selecting appropriate evaluation metrics is crucial. This study selected several important performance evaluation metrics, including Mean Absolute Error (MAE), Root Mean Squared Error (RMSE), and the coefficient of determination R^2^. MAE is the average of the absolute errors between predicted and actual values, providing an intuitive measure of the model’s performance relative to the observed values. RMSE is the square root of the average of the squared errors between predicted and actual values. R^2^ represents the proportion of the variance in the dependent variable that is predictable from the independent variables, measuring the model’s goodness of fit. In building energy consumption prediction, these metrics together provide a comprehensive evaluation framework, helping us understand the model’s performance [51]. MAE and RMSE provide direct measures of prediction error, while R^2^ provides a measure of the model’s goodness of fit. By considering these metrics together, we can more comprehensively evaluate the model’s performance and select the most suitable model for building energy consumption prediction. The formulas for these metrics are as follows:


$$MAE=\;\sum_{i=\text{1}}^{n}\frac{\left|{\hat{y}}_{i}-y_{i}\right|}{n}$$ (18)

where $y_{i}$ is the actual value, ${\hat{y}}_{i}$ is the predicted value, and n is the number of samples.


$$\begin{array}{c} RMSE=\;\sqrt{\sum {\mathrm{\backslash nolimits}}_{i=\text{1}}^{n}\frac{{\left({\hat{y}}_{i}-y_{i}\right)}^{\text{2}}}{n}} \end{array}$$ (19)

$$R^{\text{2}}=\;\frac{COV\left({\hat{y}}_{i}-y_{i}\right)}{\sqrt{Var\left(\hat{y}\right)Var(y)}}$$ (20)

These three evaluation metrics—MAE, RMSE, and R^2^—provide complementary insights into the model’s accuracy, error sensitivity, and fit. By considering all of these metrics together, we can more effectively evaluate the performance of the DNTB model in predicting chiller energy consumption.

Where i is the i-th predicted value, n is the total number of samples, $\hat{y}$ is the predicted value, and y is the actual value. COV() is the covariance function, and VAR() is the variance function.

### 4.2. Model comparative analysis

**Fig 5:**
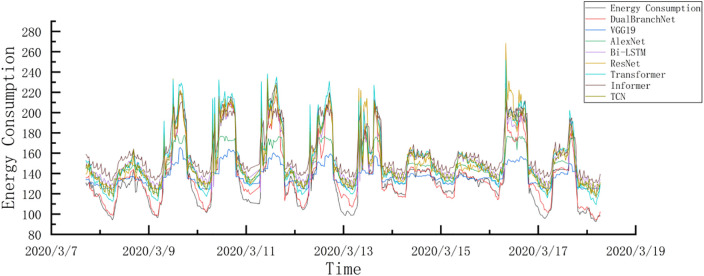
Comparison of actual chiller energy consumption vs. predicted values for different models in the long-term task.

**Fig 6:**
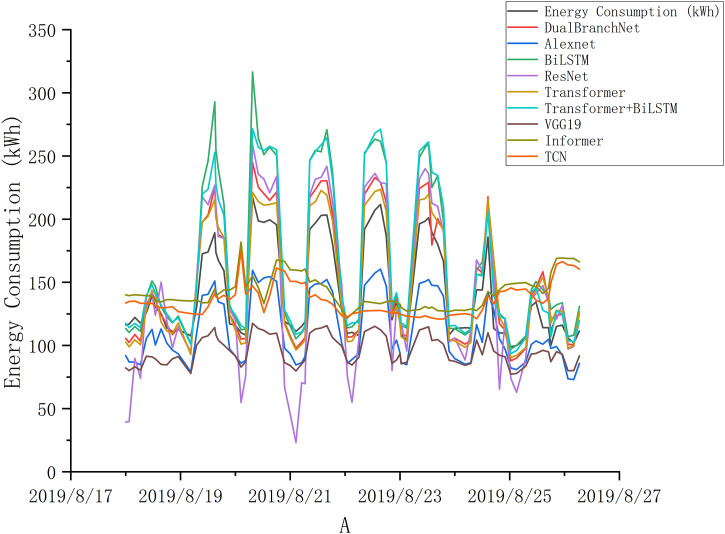
Comparison of actual chiller energy consumption vs. predicted values for different models in the short-term task.

To demonstrate the effectiveness and generalization capability of the proposed DNTB model, this study conducted extensive comparative experiments using seven different baseline models for both long-term and short-term chiller energy consumption prediction tasks. The experimental results are presented in Tables 4 and 5, as well as Figs 5 and 6.

**Table 4:** Model performance results for different models in long-term task.

Model	MSE	MAE	R2	Params
Bi-LSTM	0.0105	0.0970	0.5438	2.07MB
AlexNet	0.0106	0.0946	0.5381	12.98MB
VGG19	0.0155	0.0993	0.3265	45.54MB
ResNet	0.0119	0.0996	0.4837	60.94MB
Transformer	0.0129	0.1074	0.2863	1.02MB
Informer	0.0212	0.1336	0.2233	0.16MB
TCN	0.0153	0.1192	0.3875	0.25MB
DNTB	0.0051	0.0605	0.8031	0.63MB

**Table 5:** Model performance results for different models in short-term task.

Model	MSE	MAE	R2	Params
Bi-LSTM	0.0152	0.1032	0.3654	2.05MB
AlexNet	0.0124	0.0897	0.5066	12.98MB
VGG19	0.0197	0.119	0.2389	45.33MB
ResNet	0.0173	0.1115	−0.2891	60.94MB
Transformer	0.0151	0.1029	0.4147	1.02MB
Informer	0.0251	0.1483	0.0911	0.27MB
TCN	0.0137	0.1083	0.4064	0.25MB
DNTB	0.0080	0.0738	0.6717	0.16MB

In the long-term prediction task, among the seven baseline models, Bi-LSTM achieved the best performance, with a Mean Squared Error (MSE) of 0.0105, Mean Absolute Error (MAE) of 0.097, and an R^2^ of 0.5438. Bi-LSTM effectively handled sequential information and fit some of the chiller energy consumption data well. However, Bi-LSTM was prone to overfitting, causing the model’s performance to degrade after reaching a certain number of epochs. The worst-performing model was VGG-19, which, despite having a parameter size of 45.54MB, failed to deliver proportional improvements in performance. This suggests that overly complex models may not necessarily yield better results. Instead, they often require larger datasets to train effectively. The poor performance of VGG-19 can be attributed to its inability to capture temporal information and its inadequate fit to the chiller energy consumption data.

In the short-term prediction task, AlexNet achieved the best performance among the baseline models, with an MSE of 0.0124, MAE of 0.0897, and an R^2^ of 0.5066. The convolutional layers in AlexNet may have contributed to its ability to extract temporal features from the short-term data, positively influencing the prediction results. Similarly, VGG-19 performed the worst in this task as well, for the same reasons: its excessive complexity and inability to extract temporal features effectively. The proposed DNTB model outperformed all baseline models in both long-term and short-term prediction tasks. For long-term predictions, the DNTB model achieved an MSE of 0.0051, MAE of 0.0605, and an R^2^ of 0.8031. For short-term predictions, it achieved an MSE of 0.0084, MAE of 0.0747, and an R^2^ of 0.6538. The DNTB model demonstrated the ability to capture the temporal information of chiller energy consumption data while avoiding overfitting or underfitting, as evident from Fig 2. The Bi-LSTM branch in DNTB effectively processed sequential information and extracted relevant features, while the Transformer branch leveraged attention mechanisms to focus on features critical to the prediction task. The decision-level fusion mechanism successfully integrated the outputs of both branches, resulting in highly accurate predictions.

### 4.3. Ablation study

In order to verify the effectiveness of each module in the model, we ablated each component in the model on the short-term prediction task, including the Transformer branch, Bi-LSTM branch and Decision lever fusion modules. In the experiment of eliminating the Transformer branch, the input dimension of the Decision lever fusion module was reduced to half of the original, and the other components and parameters remained unchanged. Similarly, in the experiment of eliminating Bi-LSTM, the input module of the Decision lever fusion was also reduced to half of the original, and the other components and parameters remained unchanged. In the experiment of eliminating the Decision lever fusion module, we replaced the Decision lever fusion module with a fully connected layer (FC) and kept the other components and parameters unchanged. The results of the experiment are shown in Table 6, which shows some conclusions:

**Table 6:** Ablation study result.

Model		MSE	MAE	R2	Params
None LSTM		0.0097	0.8300	0.3606	0.12MB
None Transformer		0.0151	0.1036	0.3942	0.04MB
None Decision lever fusion	Feature level Fusion	0.0114	0.8910	0.5424	0.18MB
	Weighted Fusion	0.0742	0.2609	0.5743	0.62MB
DNTB		0.0084	0.0747	0.6538	0.16MB

**Effectiveness of the Transformer Branch:** The Transformer branch uses an attention mechanism to extract spatial and structural information from the input energy consumption data, isolating key features while reducing noise. This positively impacts prediction results.

**Importance of the Bi-LSTM Branch:** The Bi-LSTM branch is indispensable in DNTB. It captures the temporal dependencies in chiller energy consumption data, which is critical for improving model performance.

**Superiority of the Decision-Level Fusion Module:** The Decision-Level Fusion module effectively integrates features extracted by the two branches. It combines their complementary strengths to output accurate energy consumption predictions.

## 5. Conclusion

As global warming intensifies and environmental issues become increasingly critical, energy prediction and consumption reduction are gaining attention from nations and organizations worldwide. The proposed chiller energy consumption prediction model, DNTB, has potential applications in building energy management systems and energy-saving engineering projects, bridging the gap between predictive models and real-world engineering applications. This study solves the binary problem of long-term dependency and noise sensitivity through the Transformer-BiLSTM collaborative architecture, and its dynamic fusion mechanism provides a new paradigm for time series prediction. BNTB excellently models the relationship between input variables and chiller energy consumption. BNTB has shown advantages in both long-term and short-term prediction tasks. The MAE, MSE, and R2 performances for long-term prediction tasks are 0.0051, 0.0605, and 0.8031, and the MAE, MSE, and R2 performances for short-term tasks are 0.0084, 0.0747, and 0.6538, which are better than other prediction models. The proposed decision fusion lever module can better integrate the feature data of the two branches and obtain an accurate energy consumption prediction result.

The proposed DNTB model enhances the empirical model database for chiller energy consumption prediction and demonstrates strong predictive capabilities. Subsequent work will develop a quantitative version for edge devices and integrate it with the model predictive control (MPC) system to achieve closed-loop optimization. However, this study has certain limitations. The current model is trained based on tropical data, and may have performance degradation and insufficient coverage of extreme working conditions in temperate/cold regions. In the future, more data sets from different regions will be tested and Generative Adversarial Networks (GAN) will be introduced to synthesize extreme working condition data to improve this study.

## Supporting information

S1 File HVAC energy data. (XLSX)
